# When equations disagree: the impact of creatinine-based eGFR in CKD diagnosis and reclassification

**DOI:** 10.1186/s12882-025-04584-4

**Published:** 2025-11-18

**Authors:** Elena-Cristina Preda, Oana Roxana Oprea, Albert Zsolt Barabas, Ana-Maria Fotache, Minodora Dobreanu

**Affiliations:** George Emil Palade University of Medicine, Pharmacy, Science and Technology of Targu Mures, Targu Mures, Romania

**Keywords:** Chronic kidney disease, Glomerular filtration rate, Renal function tests, Precision medicine, Early diagnosis

## Abstract

Chronic kidney disease (CKD) is a major global health issue, often underdiagnosed due to its asymptomatic progression. Estimated glomerular filtration rate (eGFR) equations are essential for CKD diagnosis and management, but their performance varies across populations and age groups. This study aimed to compare three validated eGFR equations by assessing their impact on CKD classification and patient reclassification in a Romanian adult population. We retrospectively analyzed 135,630 creatinine results from adult patients admitted to a tertiary care hospital in Romania (2021–2023). eGFR was calculated using CKD-EPI 2009, CKD-EPI 2021, and European Kidney Function Consortium (EKFC) equations. Patients were categorized by age and sex, and classified according to KDIGO GFR stages. Reclassification analysis was focused on two medical decision limits: 60, 30 and 15mL/min/1.73 m². Significant differences in CKD staging were observed across equations. CKD-EPI 2021 consistently led to up-classification of GFR category, with up to 29% of patients over 40 years reclassified into less severe stages. In contrast, EKFC assigned more elderly patients, especially women over 65 years, to stage G3a. Correlation between equations ranged from moderate to strong (τ = 0.59–0.88), with better agreement in older age groups. Reclassification was most frequent around the 60 mL/min/1.73 m² threshold. The choice of eGFR equation substantially impacts CKD classification and may influence clinical decision-making and diagnosis. While EKFC and CKD-EPI 2021 correlate with CKD-EPI 2009, they produce divergent staging patterns. Further validation using measured GFR and outcome data is needed.

**Supplementary Information:**

The online version contains supplementary material available at 10.1186/s12882-025-04584-4.

## Introduction

Chronic Kidney Disease (CKD) is a widespread condition that poses a significant global health challenge, with its all-age prevalence increasing by 29% since 1990 [1]. The economic burden of kidney failure is particularly severe, especially in low- and middle-income countries. In Romania, the prevalence of CKD has been estimated at 6.74% to 7.32% [2, 3], with rates rising to approximately 25% among the elderly population [4]. The high economic burden associated with kidney failure, especially in low- and middle-income countries, underscores the importance of early detection, risk stratification, and intervention [5].

CKD often progresses silently, with diagnosis commonly delayed until advanced stages. Early identification is critical to slowing disease progression, managing metabolic complications, and reducing cardiovascular risk [6]. Serum creatinine is routinely used to estimate the glomerular filtration rate (eGFR), which, along with albuminuria, forms the basis for CKD diagnosis and staging [7].

Because serum creatinine levels can remain within normal limits despite declining kidney function, the use of eGFR equations has become essential in identifying early CKD. Over the years, several creatinine-based equations have been developed, with the CKD-EPI 2009 equation previously recommended by KDIGO [7]. However, recent guidelines allow for the use of any validated eGFR Eq. (8).

In the context of precision medicine, relying on a single eGFR equation for all patients may not be sufficient. Personalized approaches that consider demographic and biological variability are increasingly advocated for improved diagnostic accuracy.

The aim of this study was to evaluate the impact of different validated serum creatinine-based eGFR equations on CKD classification across age groups in an adult Romanian population. A secondary objective was to assess the extent of reclassification when transitioning from the CKD-EPI 2009 equation to CKD-EPI 2021 or EKFC, with a focus on medical decision limits relevant to diagnosis and management.

## Methods

### Study design and ethical approval

The retrospective observational study was conducted at the Emergency County Clinical Hospital of Targu Mures, Romania and approved by the institutional Ethics Committee (approval no.12861/28-05-2024).

### Data collection

All serum creatinine results between January 2021 and June 2023, from both inpatients and outpatients, were extracted from the medical records from the hospital database of the Emergency County Clinical Hospital of Targu Mures. Creatinine was measured using two platforms: the Roche Cobas Pro analyzer (Roche Diagnostics, Indianapolis, USA; compensated Jaffe method) and the Abbott Alinity c system (Abbott, USA; compensated alkaline picrate method). Both methods are traceable to the Isotope Dilution Mass Spectrometry (IDMS) reference standard. Additional demographic data, including sex, age, and hospital department of origin, were also collected.

The estimated glomerular filtration rate (eGFR) was calculated for each creatinine measurement using three equations: Chronic Kidney Disease Epidemiology Collaboration equation (CKD-EPI) 2009 [9], CKD-EPI 2021 [10], and the European Kidney Function Consortium (EKFC) formula [11]. For EKFC, the recommended Q-values of 0.7 mg/dL for women and 0.9 mg/dL for men were applied.

Patients were stratified into the following age groups for both sexes: [18, 25], (25, 40], (40,65], and over 65 years old, according to each equation’s breakpoint regarding age (18, 25, 40). The final group was defined based on physiological considerations, reflecting the age-associated decline in kidney function. eGFR categories were assigned according to KDIGO guidelines [7].

### Statistical analysis

eGFR values were calculated using Microsoft Excel. To evaluate the agreement between equations in assigning patients to KDIGO GFR categories, Kendall’s Tau correlation test was applied using IBM SPSS Statistics for Windows, version 20. The strength of the resulting Tau correlation coefficient (τ) was interpreted according to the thresholds proposed by Schober et al. [12], with statistical significance set at *p* < 0.05.

Reclassification analysis was conducted by comparing eGFR category assignments when switching from CKD-EPI 2009 to CKD-EPI 2021 or EKFC. Sankey diagrams illustrating reclassification patterns were generated using the online tool at www.sankeymatic.com.

The impact of reclassification was assessed in relation to three medical decision limits (MDLs):

1. 60 mL/min/1.73 m², corresponding to stage G3a and commonly used as the diagnostic threshold for CKD,
2. 30 mL/min/1.73 m², the cutoff between stages G3b and G4,where the focus shifts from risk factor modification to the management of complications and planning for renal replacement therapy,
3. 15 mL/min/1.73 m², the cutoff between stages G4 and G5, for renal replacement therapy and transplant evaluation.

The terms up-classification (overestimation) refers to patients reclassified into higher GFR categories (i.e. milder disease), while down-classification (underestimation) refers to those moved to lower GFR categories.

## Results

In this study, 135,630 serum creatinine results were analyzed, obtained from 60,323 patients (52% male, 48% female) over a three-year period. The number of measurements increased across age groups, with older age groups being more strongly represented in the dataset. The number of measurements was 3,490 for ages 18–25; 8,245 for ages 25–40; 19,565 for ages 41–65, and 33,665 for those > 65 years. A non-gaussian distribution was observed in each age group. The number of measurements, mean and median of ages, standard error and mode can be observed in supplemental material (Supplementary Table 1).

The majority of samples were collected in the emergency department (ED), particularly among younger patients (up to 75% under 25 years group) compared to the elderly (50% of the > 65 age group), whereas cardiology, internal medicine, hematology, neurology, nephrology and other departments were more represented in older age groups. The number and distribution of patients according to the hospital ward are presented in Supplementary Table 2.

eGFR estimates differed significantly between equations. In males aged 40–65, the EKFC formula classified 6.56% of patients as stage G3a, compared to 6.05% with CKD-EPI 2009 and 5.45% with CKD-EPI 2021. In the > 65 group, only 2.19% of males were classified as G1 with EKFC, versus 16.00% with CKD-EPI 2009 and 27.15% with CKD-EPI 2021. A similar shift was seen in females (Table 1).

**Table 1:** Percentage distribution of patients across KDIGO eGFR categories, stratified by age group, sex, and equation used (CKD-EPI 2009, CKD-EPI 2021, and EKFC)

		Male (%)			Female (%)		
Age group	GFR category	Epi 09	Epi 21	EKFC	Epi 09	Epi 21	EKFC
18–25	G1	89.88	91.13	77.95	93.75	94.13	85.16
	G2	6.76	5.60	18.26	3.30	2.95	11.55
	G3a	0.99	1.03	1.25	0.49	0.49	0.72
	G3b	0.47	0.34	0.65	0.43	0.40	0.43
	G4	0.26	0.26	0.22	1.32	1.35	1.35
	G5	1.64	1.64	1.68	0.72	0.69	0.80
25–40	G1	77.87	80.19	76.02	87.17	88.66	82.68
	G2	14.33	12.38	16.30	7.37	6.04	11.84
	G3a	1.94	1.76	1.88	1.12	1.04	1.16
	G3b	1.03	1.00	1.15	1.07	1.03	1.07
	G4	1.82	1.84	1.91	1.07	1.06	1.20
	G5	3.01	2.82	2.73	2.21	2.17	2.05
40–65	G1	55.64	59.86	47.18	46.55	53.62	35.08
	G2	22.07	19.36	30.00	32.75	27.01	43.04
	G3a	6.05	5.45	6.56	6.01	5.49	6.90
	G3b	4.80	4.42	5.11	4.63	4.26	4.94
	G4	4.75	4.65	4.96	3.98	3.83	4.24
	G5	6.69	6.26	6.19	6.08	5.78	5.81
> 65	G1	16.00	27.15	2.19	6.86	14.11	0.67
	G2	38.06	30.96	46.97	41.11	37.63	39.47
	G3a	14.10	12.69	16.23	14.56	13.83	18.16
	G3b	11.90	10.91	13.72	14.66	13.65	16.87
	G4	10.68	10.05	11.73	12.96	11.83	14.78
	G5	9.25	8.25	9.16	9.86	8.95	10.06

Epi 09–2009 Chronic Kidney Disease Epidemiology Collaboration equation, Epi 21–2021 Chronic Kidney Disease Epidemiology Collaboration equation, EKFC- European Kidney Function Consortium equation

Correlation between equations was assessed using Kendall’s Tau coefficient. The strongest agreement was observed between CKD-EPI 2009 and CKD-EPI 2021 (τ > 0.9 in all age groups). The agreement between CKD-EPI 2021 and EKFC was moderate to strong (τ = 0.60–0.88), particularly in younger patients (Table 2). All correlations were significant, with *p* < 0.01.

**Table 2:** Agreement between eGFR equations in classifying patients into KDIGO GFR stages: kendall’s Tau coefficients by age group and sex

		Male											
		18–25 y.o.			25–40 y.o.			40–65 y.o.			> 65y.o.		
		Epi 09	Epi 21	EKFC	Epi 09	Epi 21	EKFC	Epi 09	Epi 21	EKFC	Epi 09	Epi 21	EKFC
Female	Epi 09	-	0.91 (0.88, 0.94)	0.67 (0.65, 0.70)	-	0.88 (0.87, 0.90)	0.80 (0.79, 0.82)	-	0.94 (0.94, 0.95)	0.90 (0.89, 0.91)	-	0.91 (0.90, 0.92)	0.89 (0.88, 0.91)
	Epi 21	0.93 (0.90, 0.91)	-	0.70 (0.68, 0.73)	0.85 (0.83, 0.86)	-	0.74 (0.72, 0.75)	0.91 (0.91, 0.92)	-	0.86 (0.82, 0.87)	0.92 (0.92, 0.93)	-	0.87 (0.86, 0.88)
	EKFC	0.56 (0.53, 0.60)	0.59 (0.56, 0.62)	-	0.66 (0.64, 0.67)	0.60 (0.58, 0.61)	-	0.88 (0.87, 0.89)	0.82 (0.81, 0.83)	-	0.91 (0.90, 0.91)	0.88 (0.87, 0.88)	-

Gray cells contain Tau coefficients for female analysis. y.o. – years old, Epi 09–2009 Chronic Kidney Disease Epidemiology Collaboration equation, Epi 21–2021 Chronic Kidney Disease Epidemiology Collaboration equation, EKFC- European Kidney Function Consortium equation

**Fig. 1:**
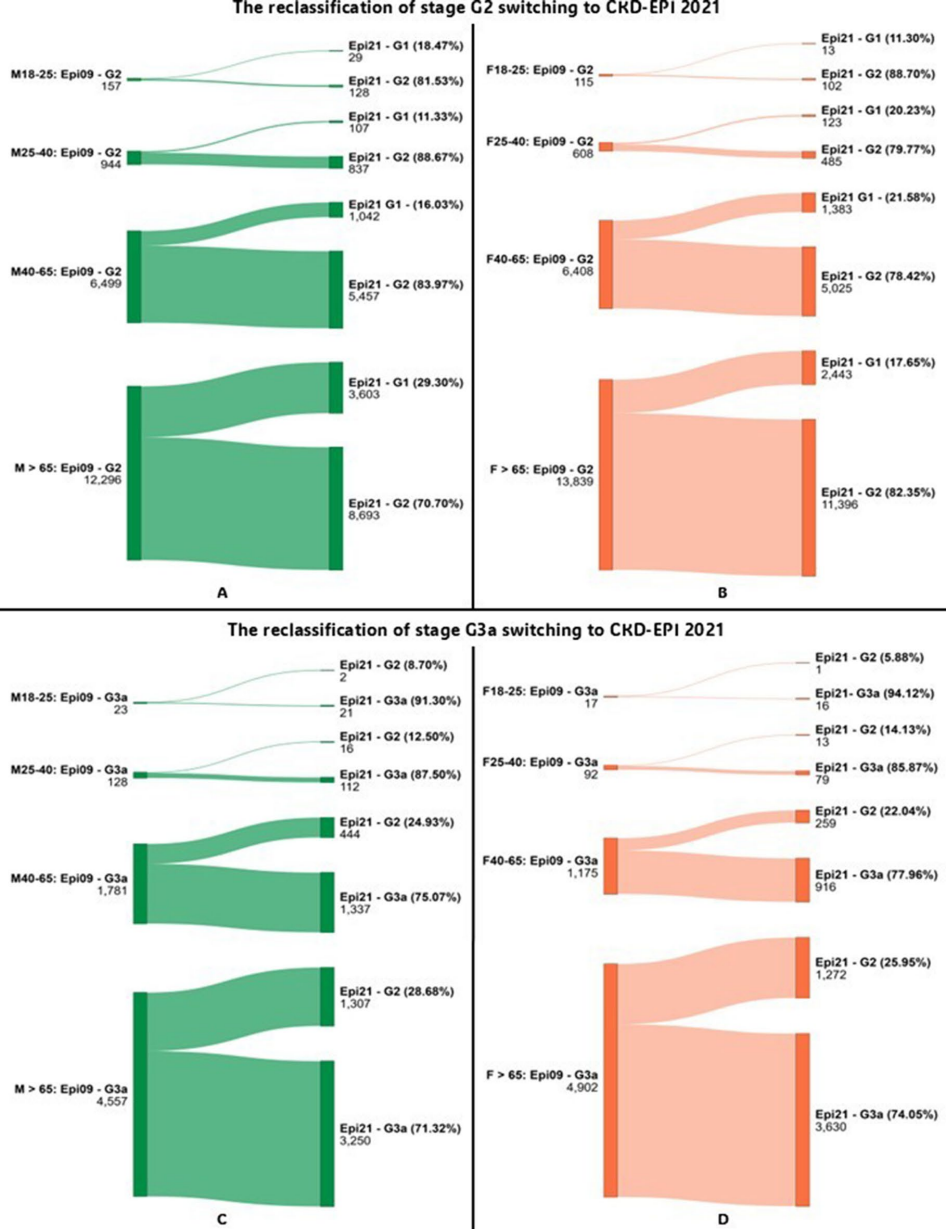
- Diagram showing reclassification of patients between CKD stages for G2 and G3a when switching from CKD-EPI 2009 to CKD-EPI 2021, by sex. Abbreviations: Epi 09–2009 Chronic Kidney Disease Epidemiology Collaboration equation; Epi 21–2021 Chronic Kidney Disease Epidemiology Collaboration equation; EKFC – European Kidney Function Consortium equation

**Fig. 2:**
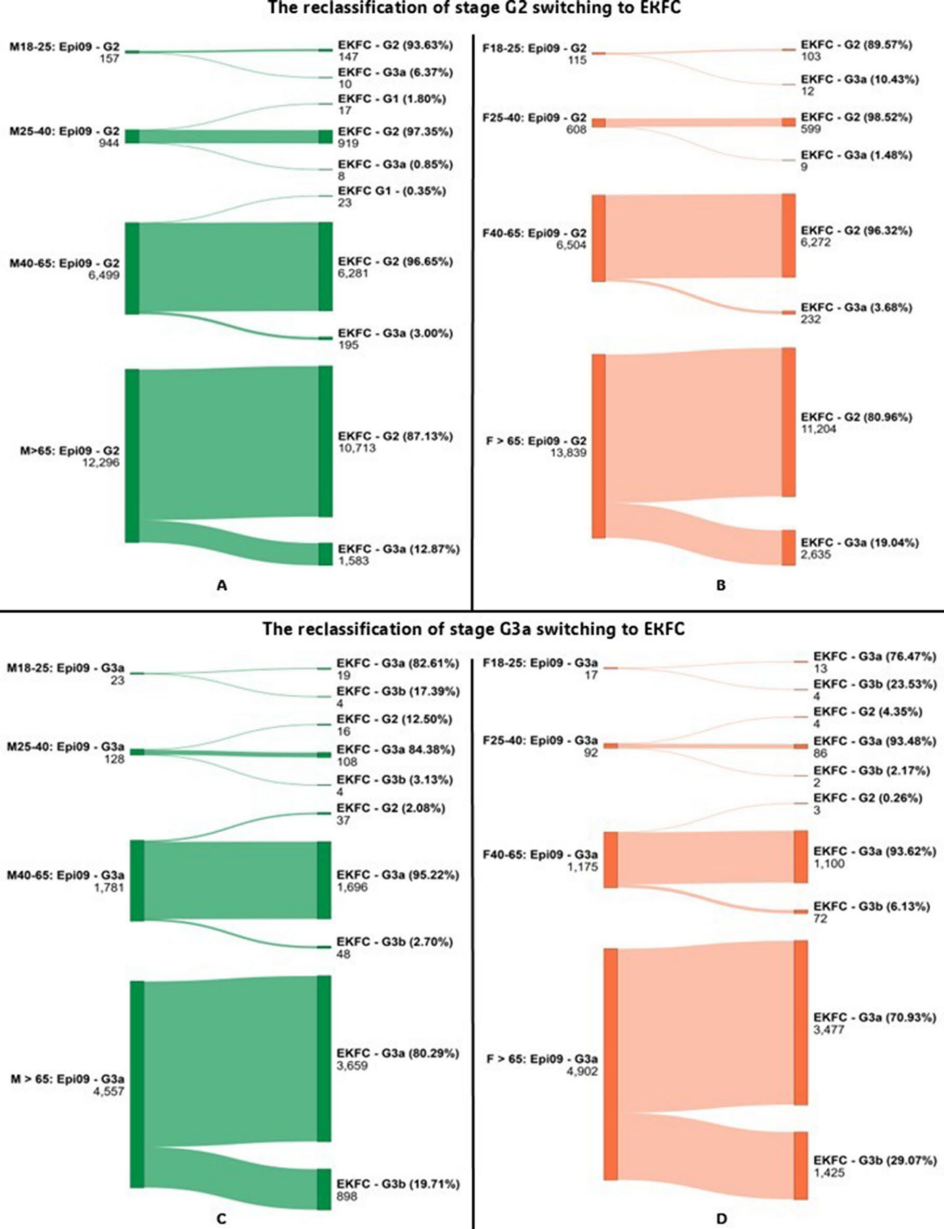
- Diagram showing reclassification of patients between CKD stages for G2 and G3a when switching from CKD-EPI 2009 to EKFC, by sex. Abbreviations: Epi 09–2009 Chronic Kidney Disease Epidemiology Collaboration equation; Epi 21–2021 Chronic Kidney Disease Epidemiology Collaboration equation; EKFC – European Kidney Function Consortium equation

**Fig. 3:**
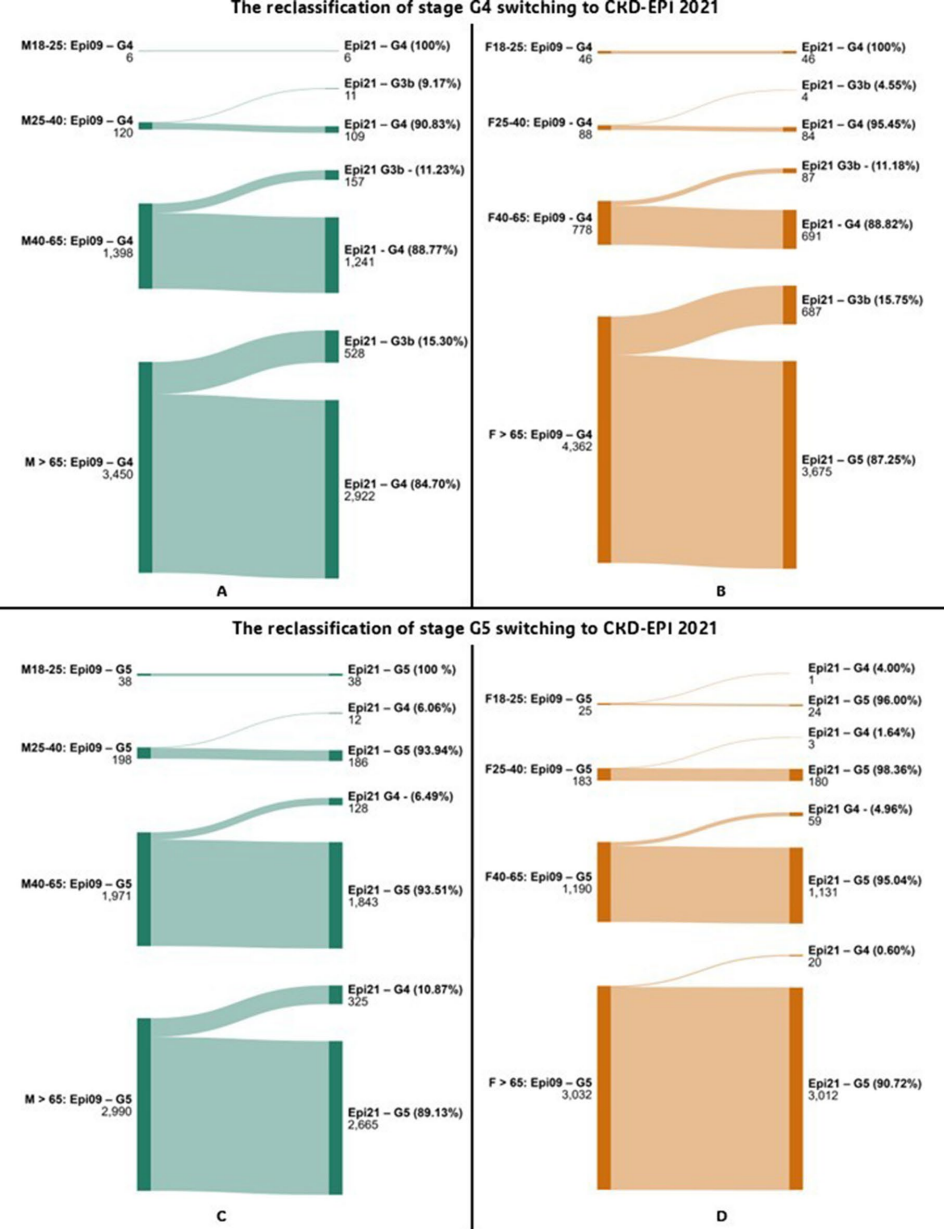
- Diagram showing reclassification of patients between CKD stages for G4 and G5 when switching from CKD-EPI 2009 to CKD-EPI 2021, by sex. Abbreviations: Epi 09–2009 Chronic Kidney Disease Epidemiology Collaboration equation; Epi 21–2021 Chronic Kidney Disease Epidemiology Collaboration equation; EKFC – European Kidney Function Consortium equation

**Fig. 4:**
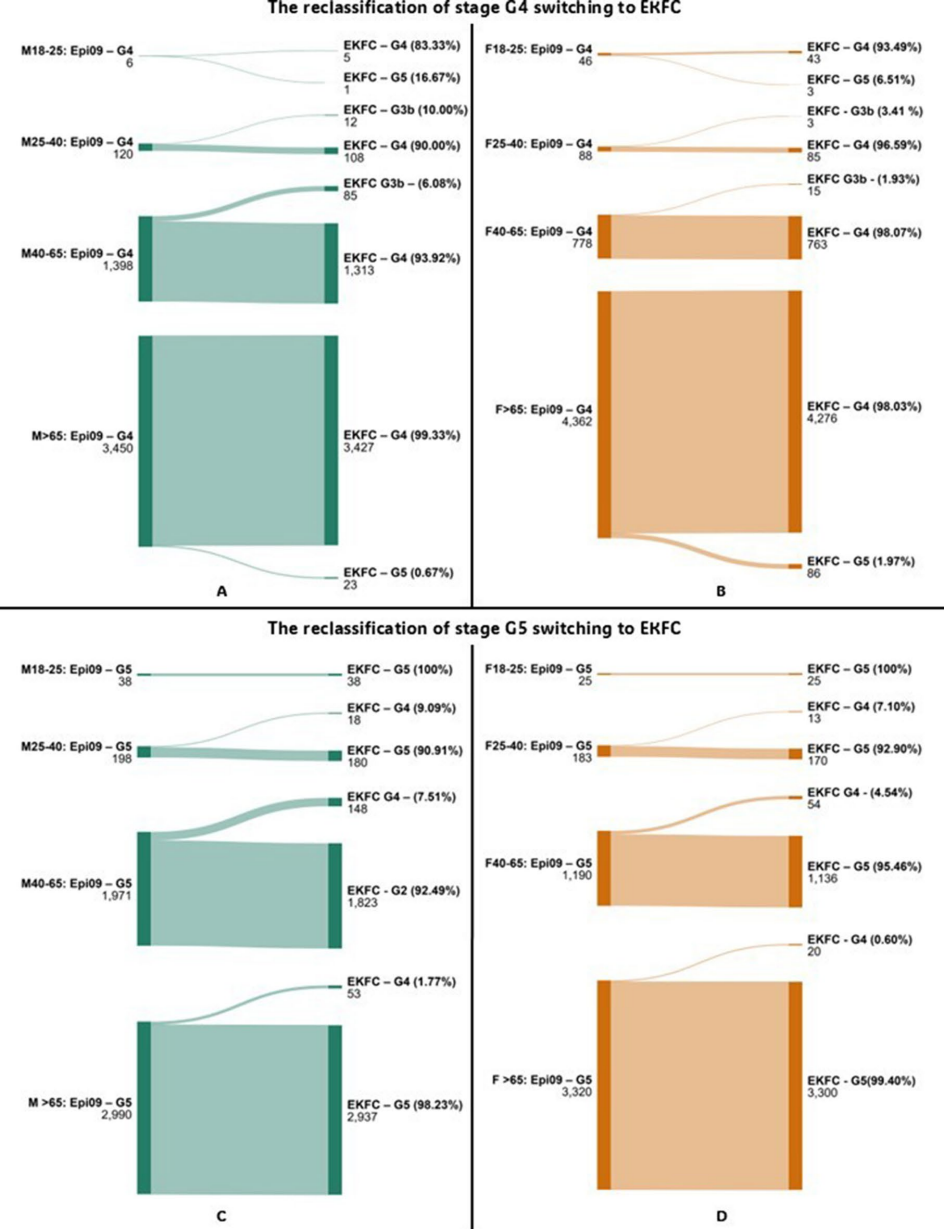
- Diagram showing reclassification of patients between CKD stages for G4 and G5 when switching from CKD-EPI 2009 to EKFC, by sex. Abbreviations: Epi 09–2009 Chronic Kidney Disease Epidemiology Collaboration equation; Epi 21–2021 Chronic Kidney Disease Epidemiology Collaboration equation; EKFC – European Kidney Function Consortium equation

Reclassification analysis showed that transitioning from CKD-EPI 2009 to EKFC or CKD-EPI 2021 led to notable changes in patient categorization, especially near key medical decision limits (MDLs). For instance, in the 25–40 age group, the EKFC equation reclassified 10.00% of male patients from stage G4 to G3b, and 9.09% from G5 to G4—suggesting a lower estimated disease severity. Similarly, substantial reclassification was observed from G2 to G3a in patients over 65 years groups, with EKFC identifying more patients with reduced renal function compared to CKD-EPI 2009. Sankey diagrams illustrating reclassification between G2 and G3a stages are shown in Figs. 1 and 2. Detailed data on reclassification of G3b stage is presented in Table 3. Full reclassification data for G4 and G5 stages are provided in Figs. 3 and 4.

**Table 3:** Reclassification patterns of CKD patients in stages G3b when switching from CKD-EPI 2009 to CKD-EPI 2021 and EKFC equations, by age and sex

Age Group	Sex	CKD-CKD-EPI 2009	CKD-EPI 2021		EKFC	
			G3b	Reclassification Stage: n (%)	G3b	Reclassification Stage: n (%)
18–25	**M**	11	8	↑ G3a: 3 (27.27)	11	NA
	**F**	15	14	↑ G3a: 1 (6.67)	11	↓ G4: 4 (26.67)
25–40	**M**	68	55	↑ G3a: 13 (19.12)	60	↑ G3a: 8 (11.76)
	**F**	88	85	↑ G3a: 3 (3.41)	87	↓ G4: 1 (1.14)
40–65	**M**	1431	1145	↑ G3a: 268 (18.97)	1371	↑ G3a: 42 (2.97)
	**F**	905	746	↑ G3a: 159 (17.57)	879	↑ G3a: 13 (1.44), ↓ G4: 13 (1.44)
> 65	**M**	3846	2997	↑ G3a: 849 (22.07)	3534	↓ G4: 312 (8.11)
	**F**	4934	3908	↑ G3a: 1026 (20.79)	4253	↓ G4: 681 (13.80)

## Discussion

This study evaluated the classification patterns of three creatinine-based equations for eGFR - CKD-EPI 2009, CKD-EPI 2021, and EKFC across different age groups in a Romanian population. Although no equation can universally meet the needs of all clinical contexts, understanding how they differ in classifying patients into CKD stages is essential for optimizing detection and management strategies.

Main findings of the study revealed (1) that CKD-EPI 2021 equation consistently resulted in up-classification compared to CKD-EPI 2009, especially in individuals over 40 years of age and (2) most patients were classified into stage G3a using EKFC.

In our study, most of the patient’s samples were collected in the emergency department (ED), with a higher proportion among younger patients compared to the elderly. The ED handles a large number of patients, which makes it important for CKD screening [13]. In Nigeria, a study showed that following the ED admission, 74% of patients were firstly diagnosed with CKD [14].

### Switching from 2009 CKD-EPI to 2021 CKD-EPI

On the premise that race is a social concept rather than a biological one, the 2021 CKD-EPI equation is the first to remove the race coefficient. While this equation performs better for African-American patients in the U.S. compared to the previous version, it tends to overestimate eGFR in the rest of patients [15]. In Europe, its performance is suboptimal for the Caucasian population, with only minor improvements for black patients [16].

Among the three equations, CKD-EPI 2021 identified the smallest proportion of patients in G3a, G3b, G4 and G5 especially in individuals over 40 years old. The correlation observed between the CKD-EPI 2009 and CKD-EPI 2021 equations in classifying patients into identical glomerular filtration rate (GFR) categories demonstrated strong to very strong agreement across all age groups, with τ coefficients exceeding 0.85.

Despite the strong agreement between the two equations, when transitioning from the 2009 version to the 2021 one, we observe a constant up-classification trend across all age groups in our study. This trend may delay CKD diagnosis and nephrology referral, particularly around the MDL of 60 mL/min/1.73 m² that separates stages G2 and G3a. For example, in our cohort, up to 29% of patients no longer met CKD criteria when reclassified using the 2021 equation. Similarly, among patients over 40 years old initially classified in stage G4, between 11 and 15% of male and female were reclassified to stage G3b, potentially affecting nephrologist referral and renal replacement planning.

These findings align with previous studies conducted in Caucasian populations. In a similar study conducted in Canada on a Caucasian population, 28% of patients were reclassified from G5 to G4 using CKD-EPI 2021 [17], while a Swedish study reported a 17% shift in the same direction with many additional patients (39%) reclassified into stage G2 and therefore no longer considered to have CKD [18]. The reclassified patients had higher risks for all cause death and mortality for cardiovascular disease (CVD) [18]. However, the classification has not been evaluated according to age distribution.

### Switching from the 2009 CKD-EPI to EKFC

Similarly to 2021 CKD-EPI, the European Kidney Function Consortium (EKFC) does not include a race coefficient [11]. Developed using data predominantly from European populations [19], the EKFC equation includes a “Q value” representing the mean serum creatinine level in the derivation cohort [11]. This adaptable Q value has demonstrated the equation’s feasibility, even outside Europe, including in African cohorts [20].

In our analysis, agreement between CKD-EPI 2009 and EKFC in classifying patients into similar GFR categories ranged from moderate to strong in adults under 40, with lower correlations observed in female patients (τ = 0.56 and 0.66 for females vs. 0.67 and 0.80 for males). In contrast, in patients over 40, correlations exceeded τ = 0.88 in both sexes, indicating strong concordance. In a study evaluating concordance and discrepancies among creatinine based eGFR equations in Swedish patients over 60 years old, the correlation between CKD-EPI 2009 and EKFC was poor (Cohen K 0.54) [21].

Transitioning from the CKD-EPI 2009 to the EKFC equation in adults, no consistent directional pattern was observed across all age groups. Notable shifts occurred around key MDLs, at the GFR threshold of 60 mL/min/1.73 m², the EKFC equation exhibited a trend towards down-classification, from G2 to the G3a category, leading to an increased number of patients meeting the diagnostic criteria for CKD. The most significant reclassification occurred in patients over 65 years, especially women, with 19% women and 13% men being reclassified from G2 to G3a, and 13% women and 8% men in the same age group being reclassified from G3b to G4, increasing CKD diagnosis and nephrologist referral. These differences may reflect sex-specific patterns in age-related GFR decline, as suggested by previous studies [22]. At the 15 mL/min/1.73 m² MDL, moderate proportions of patients (less than 9% in each age group) were reclassified to milder GFR stages.

Similar findings have been reported in other studies. An analysis conducted in an American population demonstrated increased CKD prevalence when applying the EKFC Eq. [23], while an Italian study reported comparable patterns of down-classification at early CKD stages [24]. In older adults in Sweden the CKD-EPI 2009 equation showed higher estimates of GFR compared to EKFC [21]. In the northern European population, Russel, et al., showed that the reclassified patients had higher risk of all cause mortality [25]. These observations suggest that EKFC may enhance CKD identification in European populations, particularly in older adults, though further evaluation using measured GFR and clinical outcomes is needed.

### 2021 CKD-EPI vs. EKFC

When stratified by sex, the agreement between EKFC and CKD-EPI 2021 equations showed notable variability. In female patients, Kendall’s Tau values ranged from 0.59 to 0.88, while in males the correlation was somewhat higher and more consistent (τ = 0.70–0.88). In both sexes, the strongest agreement was observed in individuals over 65 years of age. These findings suggest that while the two equations yield increasingly concordant classifications with advancing age, discrepancies are more pronounced in younger populations, particularly among women. Although the EKFC and CKD-EPI 2021 equations demonstrated a strong correlation in terms of patient ranking by eGFR (τ = 0.60–0.88), they yielded notably different absolute estimates. As a result, EKFC classified a significantly higher number of patients into stage G3a, especially in individuals over 65 years of age. This apparent paradox - strong statistical agreement alongside substantial reclassification - reflects the fact that correlation coefficients assess the relative ordering of patients but not the absolute eGFR values or threshold-based categorization. Therefore, even a strong correlation may mask clinically meaningful differences in staging and potential treatment implications.

Accurate assessment of kidney function is essential for precision medicine CKD. The EKFC equation demonstrates improved performance in older adults compared with the CKD-EPI 2009, due to its development EKFC included several older adult cohorts [9, 11]. Its use is therefore preferable in patients aged > 65 years, ensuring more reliable GFR estimation and optimized clinical decision-making.

These findings must also be interpreted in light of the current international recommendations. Due to the varying performance of creatinine-based equations across populations, the latest KDIGO guideline [8] recommends using an equation validated for the target population. In the United States, the race-free 2021 CKD-EPI is now the standard. However, European bodies such as the European Federation of Clinical Chemistry and Laboratory Medicine (EFLM) and the European Renal Association (ERA) have expressed reservations regarding its applicability to European populations. While acknowledging the promise of the EKFC equation, both organizations recommend awaiting further evidence before endorsing its widespread clinical use [26–28].

## Conclusion

In this Romanian cohort, the choice of eGFR equation had a substantial impact on CKD staging. Transitioning from CKD-EPI 2009 to either CKD-EPI 2021 or EKFC resulted in clinically relevant reclassifications, particularly around MDL of 60, 30 and 15 mL/min/1.73 m². CKD-EPI 2021 generally led to up-classification of the GFR category, reducing the number of patients identified with CKD, while EKFC tended to reclassify more patients into stage G3a, especially in adults over 65 years of age.

These reclassifications may influence clinical decisions such as timing of nephrology referral, risk stratification, or eligibility for interventions like renal replacement therapy. Although the equations showed strong statistical correlation, their divergent classification patterns emphasize the need for careful consideration when implementing new formulas in clinical practice.

## Limitations

This study has several limitations. First, it relied exclusively on estimated GFR equations without access to measured GFR, albuminuria or clinical outcome data, limiting the ability to assess true diagnostic accuracy or prognostic value. Second, single-center design and the cross-sectional nature of the dataset restrict generalizability and preclude any evaluation of longitudinal trends in renal function.

Additionally, young adults—particularly those aged 18–25—were underrepresented, despite being a key group where differences in equation performance may be most relevant. Lastly, although creatinine measurements were IDMS-traceable, they were obtained from two different analytical platforms, which may introduce minor inter-method variability.

## Supplementary Information

Below is the link to the electronic supplementary material.

Supplementary Material 1

Supplementary Material 2

## Data Availability

The datasets used and/or analysed during the current study are available from the corresponding author on reasonable request.

## Abbreviations

CKD: Chronic kidney disease
eGFR: Estimated glomerular filtration rate
EKFC: European Kidney Function Consortium
IDMS: Isotope Dilution Mass Spectrometry
MDL: Medical decision limits
CKD-EPI: Chronic Kidney Disease Epidemiology Collaboration equation
