# Supplementary material for: When equations disagree: the impact of creatinine-based eGFR in CKD diagnosis and reclassification
Source: BMC Nephrol. 2025 Nov 18;26:647. doi: 10.1186/s12882-025-04584-4 (PMC12624983; doi:10.1186/s12882-025-04584-4)
Supplement: Supplementary file 1 — Supplementary Material 1 [file 12882_2025_4584_MOESM1_ESM.docx]

Supplementary Table 2. Creatinine measurements number and distribution according to the hospital ward of origin.

|  | 18-25 y.o. | | 25-40 y.o. | | 40-65 y.o. | | >65 y.o. | |
| --- | --- | --- | --- | --- | --- | --- | --- | --- |
| Department | M n(%) | F n(%) | M n(%) | F n(%) | M n(%) | F n(%) | M n(%) | F n(%) |
| ED | 1747 (75.2) | 2204 (63.2) | 4569 (69.3) | 4229 (51.3) | 14979 (50.9) | 10533 (53.8) | 14699 (45.5) | 16177 (48.1) |
| Surgery | 203 (8.7) | 111 (3.2) | 729 (11.1) | 347 (4.2) | 4229 (14.4) | 2240 (7.6) | 4228 (13.1) | 3312 (9.8) |
| Cardiology | 38 (1.6) | 25 (0.7) | 227 (3.4) | 69 (0.8) | 3169 (10.8) | 1332 (4.5) | 3238 (10.0) | 2870 (8.5) |
| Internal medicine | 17 (0.7) | 12 (0.3) | 84 (1.3) | 87 (1.1) | 1346 (4.6) | 915 (3.1) | 3314 (10.3) | 3958 (11.8) |
| Hematology | 77 (3.3) | 22 (0.6) | 155 (2.4) | 155 (1.9) | 526 (1.8) | 587 (2.0) | 673 (2.1) | 700 (2.1) |
| Neurology | 17 (0.7) | 38 (1.1) | 101 (1.5) | 108 (1.3) | 1552 (5.3) | 443 (1.5) | 1720 (5.3) | 1430 (4.3) |
| Nephrology | 24 (1.0) | 21 (0.6) | 142 (2.2) | 150 (1.8) | 789 (2.7) | 475 (1.6) | 998 (3.1) | 1131 (3.4) |
| Gynecology | NA | 835 (23.9) | NA | 2430 (29.5) | NA | 622 (2.1) | NA | 240 (1.0) |
| Other departments * | 199 (8.6) | 222 (6.4) | 582 (8.8) | 670 (8.1) | 2856 (9.7) | 2418 (23.7) | 3438 (10.6) | 3847 (11.4) |

*Other departments include Outpatient Clinics, the ICU, Rehabilitation Medicine, and other smaller departments. y.o.-Years old, ED- emergency department
