# Supplementary material for: When equations disagree: the impact of creatinine-based eGFR in CKD diagnosis and reclassification
Source: BMC Nephrol. 2025 Nov 18;26:647. doi: 10.1186/s12882-025-04584-4 (PMC12624983; doi:10.1186/s12882-025-04584-4)
Supplement: Supplementary file 2 — Supplementary Material 2 [file 12882_2025_4584_MOESM2_ESM.docx]

Supplementary Table 1. Descriptive statistics for the age and sex groups. The table contains information about mean, median, standard error, most frequent age value in the group (mode), and Inter-Quartile Range (IQR).

| **Sex** | **Age group** | **Number of measurements** | **Age Mean** | **Age Median** | **Age Standard Error** | **Age Mode** | **Age IQR** |
| --- | --- | --- | --- | --- | --- | --- | --- |
| **Male** | **18-25** | 2322 | 22 | 22 | 0.05 | 20 | 20-23 |
|  | **26-40** | 6589 | 34 | 34 | 0.05 | 40 | 30-37 |
|  | **40-65** | 29446 | 55 | 55 | 0.04 | 64 | 49-61 |
|  | **>65** | 32308 | 75 | 74 | 0.04 | 67 | 70-80 |
| **Female** | **18-25** | 3490 | 21 | 21 | 0.04 | 19 | 19-23 |
|  | **25-40** | 8245 | 33 | 33 | 0.05 | 34 | 29-37 |
|  | **40-65** | 19565 | 55 | 55 | 0.05 | 65 | 49-61 |
|  | **>65** | 33665 | 77 | 76 | 0.04 | 75 | 70-78 |
